# Correction to: Development and Validation of a Nonremission Risk Prediction Model in First-Episode Psychosis: An Analysis of 2 Longitudinal Studies

**DOI:** 10.1093/schizbullopen/sgac070

**Published:** 2022-11-30

This is a correction to: Samuel P Leighton, Rajeev Krishnadas, Rachel Upthegrove, Steven Marwaha, Ewout W Steyerberg, Georgios V Gkoutos, Matthew R Broome, Peter F Liddle, Linda Everard, Swaran P Singh, Nicholas Freemantle, David Fowler, Peter B Jones, Vimal Sharma, Robin Murray, Til Wykes, Richard J Drake, Iain Buchan, Simon Rogers, Jonathan Cavanagh, Shon W Lewis, Max Birchwood, Pavan K Mallikarjun, Development and Validation of a Nonremission Risk Prediction Model in First-Episode Psychosis: An Analysis of 2 Longitudinal Studies, *Schizophrenia Bulletin Open*, Volume 2, Issue 1, January 2021, sgab041, https://doi.org/10.1093/schizbullopen/sgab041

In this article, during the analysis we combined the data across the 10 multiple imputations into a single dataset from which we determined the C-statistic performance, and calibration intercept and slope, also presented in figure 2. This resulted in correct estimates of the values but narrower confidence intervals than if we had correctly applied Rubin’s Rules.^1^ The correct confidence intervals for the internal validation C-statistic and calibration slope are 0.74 (0.72, 0.76) and 0.84 (0.76, 0.92), respectively. The correct confidence intervals for the external validation C-statistic, calibration intercept and slope are 0.73 (0.64, 0.81), 0.12 (-0.20, 0.43) and 0.98 (0.53, 1.44). The significance and interpretation of the results are unchanged. The original and updated R code are available online (https://github.com/samleighton87/NEDEN_Outlook_FEP).

These details have been corrected only in this correction notice to preserve the published version of record.
